# Retraction: Intervention effect of rehabilitation robotic bed under machine learning combined with intensive motor training on stroke patients with hemiplegia

**DOI:** 10.3389/fnbot.2026.2023207

**Published:** 2026-10-14

**Authors:** Frontiers Editorial Office

The journal retracts the 09 June 2022 article cited above.

Following publication, concerns were raised regarding the ethical approval for this study. The article states that ethical approval was granted by an institution that the authors are not affiliated with (“Medical Ethics Committee of Ganzhou Hospital”).

An investigation was conducted in accordance with Frontiers' policies. The authors did not respond to queries after publication, and the publisher was unable to verify the existence of Ganzhou Hospital or confirm valid ethical approval for the research. Lack of appropriate ethical approval is a breach of Frontiers' guidelines and those of the Committee on Publication Ethics. As such, this article is being retracted.

This retraction was approved by the Chief Editors of Frontiers in Neurorobotics and the Chief Executive Editor of Frontiers. The authors received a communication regarding the retraction and had a chance to respond. This communication has been recorded by the publisher.

Frontiers would like to thank the users on PubPeer for bringing the published article to our attention.

